# Cytotoxic and apoptogenic effects of *Dracocephalum kotschyi* Boiss., extracts against human glioblastoma U87 cells

**Published:** 2020

**Authors:** Mahmoud Shaabani, Seyed Hadi Mousavi, Majid Azizi, Ali Ashraf Jafari

**Affiliations:** 1 *Department of Horticulture Science and Agronomy, Science and Research Branch, Islamic Azad University, Tehran, Iran*; 2 *Medical Toxicology Research Center, Faculty of Medicine, Mashhad University of Medical Sciences, Mashhad 917794-8564, Iran*; 3 *Department of Horticulture, College of Agriculture, Ferdowsi University of Mashhad, Mashhad, Iran*; 4 *Research Institute of Forests and Rangelands, Agricultural Research, Education and Extension Organization (AREEO), Tehran, Iran *

**Keywords:** Dracocephalum kotschyi Boiss. GC/MS, Glioblastoma, Oxidative stress

## Abstract

**Objective:**

Glioblastoma multiforme (GBM) is the most aggressive and malignant brain tumor and has a poor prognosis. This study was aimed to investigate the cytotoxic effects of *Dracocephalum kotschyi* Boiss.** (***D. kotschyi*) extracts in GBM U87 cell line.

**Materials and Methods:**

The extracts of *D. kotschyi* obtained by two different ways of Soxhlet and soaked. The cytotoxic effects of *D. kotschyi* extracts were measured using MTT assay following treatment for different times of exposure (24, 48, and 72 hr) and at different concentrations of *D. kotschyi* extracts. The effects of *D. kotschyi* extracts on cellular oxidative stress were also evaluated by measuring cellular ROS levels. Furthermore, cellular death and apoptosis were studied by sub G1 analysis and Annexin V-FITC/propidium iodide (PI) staining using flow cytometry method, respectively. Characterization of the extracts was carried out using gas chromatography/mass spectrometry (GC/MS) analysis by Agilent GC-MSD system.

**Results:**

Our results indicated that *D. kotschyi* extracts decreased U87 cell viability in a time- and dose-dependent manner. Moreover, treatment with *D. kotschyi* extracted by Soxhlet for 24 and 48 hr significantly increased the levels of cellular ROS and Sub G1 population (p<0.001-0.05 for all cases). Furthermore, GC/MS analysis revealed that essential oils of *D. kotschyi* mainly consisted of β-caryophellene, α-pinene and limonene.

**Conclusion:**

Our findings demonstrated that *D. kotschyi* extracts can exert cytotoxic effects against GBM U87 cell line in a time- and concentration-dependent manner, and these effects may be mediated through intracellular ROS accumulating. However, further studies should be performed to confirm the efficacy and exact mechanism of action of the extracts.

## Introduction

Glioblastoma multiforme (GBM) is the most aggressive, lethal, and highly recurrent type of tumors of the central nervous system (Metz et al., 2016 ▶; Rahimi et al., 2019a ▶). Patients diagnosed with GBM have a poor prognosis with an average survival time of 12 to 15 months (Rahimi et al., 2019a ▶; Yin et al., 2019 ▶). GBM can arise at any age but the incidence is higher among 55 to 65-year-old individuals (Davis, 2016 ▶). Despite novel improvements in therapeutic strategies including surgery, radiation therapy, and chemotherapy, the overall survival rate of GBM patients remains low due to tumor recurrence, drug resistance and unexpected side effects. 

Many synthetic anti-cancer agents have been studied in recent years, but no medication has been found to be well tolerated and fully effective. The toxicity and complication of chemotherapeutic agents have been reported as major problems in cancer therapy, supporting the need for detection of new natural products to improve the efficacy of current treatment modalities. As compared with the current anti- cancer modalities, natural therapies such as using medicinal herbs are considered more effective strategies in cancer therapy because of their safety, low-cost, and low toxic effects (Seca and Pinto, 2018 ▶). Medicinal herb extractss contain certain phytochemicals which are beneficial for treatment and management of cancers and exert their effects through inducing tumor cell death or stimulating immune responses (Yin et al., 2017 ▶).

*Dracocephalum kotschyi* Boiss. is a wild-growing flowering plant that belongs to the Lamiaceae family (Sadraei et al., 2015a ▶). Several studies in Iranian ancient medicine about *D. kotschyi*, recognized several medicinal activities including anti-hyperlipidemia, antispasmodic, analgesic, and anti-tumor properties of the plant (Kalantar et al., 2018 ▶). Moreover, it was also reported that *D. kotschyi* has several therapeutic effects against digestive disorders and rheumatoid diseases. Recent studies revealed that *D. kotschyi* extracts contain some compositions which have great anti-cancer activities but the exact cellular mechanism by which *D. kotschyi* induced cell death, has not been explored (Jahaniani et al., 2005 ▶). Therefore, the present study aimed to examine whether *D. kotschyi* can induce cellular death and inhibit the growth of U87 glioma cells. 

## Materials and Methods

**Chemicals** **and** **reagents**

Roswell Park Memorial Institute (RPMI)-1640, Dulbecco’s Modified Eagle Medium (DMEM), fetal bovine serum (FBS), penicillin (50 IU/ml) and streptomycin (50 μg/ml) were purchased from Gibco (Gaithersburg, MD). WST-1 assay kit was from Roche Diagnostic, Mannheim, Germany. Other laboratory chemicals were obtained from Sigma-Aldrich (Zwijndrecht, Netherlands).

**Preparation of** ***D. kotschyi*** **extracts**

*D.* *kotschyi* was collected from Qouchan (Khorasan Razavi province, Iran) heights (37° 06′ 22.4″N and 58° 30′ 34.12″ E), identified and registered with No. MSH131 by Botanical Research Center of Qouchan Payame Noor University. The plant samples were air-dried and milled at room temperature. The extracts were then prepared by two Soxhlet and soaked methods using ethanol (70% v/v) for 48 hr. Afterward, the solvent was removed by rotary-evaporation at 37°C and dried by freeze drying (Rahimi et al., 2018a ▶, 2018b ▶, 2019b ▶). 

**Cell culture**

U87 cells were purchased from Pasture institute (Tehran, Iran). The cells were grown in DMEM medium supplemented with 10% v/v FBS and 1% v/v streptomycin/penicillin and maintained at 37°C in 5% v/v CO_2_ humidified atmosphere (Askari et al., 2016b ▶).

**Growth inhibition studies**

The 3-(4, 5-dimethylthiazol-2-yl)-2, 5-diphenyltetrazolium bromide (MTT) tetrazolium colorimetry assay was performed to investigate the cytotoxic effects of *D. kotschyi* on glioblastoma cells. Briefly, U87 cells were seeded in 96-well plate and treated with Soxhlet and soaked extracts of *D. kotschyi* (0-1600 µg/ml) for 24, 48 and 72 hr. Following treatment, 20 μl of the MTT solution (5 mg/ml) was added to each well. Plates were incubated in the dark at 37°C for 3 hr. Next, the medium was removed and DMSO (100 μl) was added to dissolve the formazan crystals produced by viable cells. The absorbance was measured at 545-630 nm using an ELISA reader (Start Fax 2100; Awareness Technology Inc.) (Askari and Shafiee-Nick, 2019b ▶, 2019c ▶). Mean of three independent experiments carried out at least in triplicate, is reported. The IC_50_ value was measured as doses which resulted in 50% reduction of the number of viable cells (Askari et al., 2016a ▶, 2019b ▶, 2019c ▶). The extracts was dissolved in DMSO 50 mg/ml (stock) where the final concentration of DMSO was lower than 0.1% v/v. Noteworthy, control group received.

**Flowcytometric analysis and quantification of apoptosis**

The cell cycle distribution was studied by flow cytometric analysis using propidium iodide (PI) staining method. Briefly, U87 cells were seeded in a 6-well plate overnight and then treated with fresh medium containing 0-1600 µg/ml *D. kotschyi* for 24 and 48 hr. Treated cells were harvested with trypsin and fixed with 70% cold ethanol for 2 hr at 4°C. Finally, the fixed cells were incubated with RNase at 37°C for 30 min and then stained with PI for 30 min. The stained cells were analyzed by FACSCalibur flow cytometer (BD Biosciences, USA) and Flowjo software (Tree Star Inc.). Each experiment was performed in triplicate (Kianmehr et al., 2017 ▶; Marjaneh et al., 2018 ▶).

Next, we investigated the apoptotic effects of *D. kotschyi* on glioma cells using Annexin V/PI apoptosis assay kit (Cayman Chemical, Ann Arbor, MI). In brief, U87 cells were treated with two different concentrations (200 and 400 µg/ml) of *D. kotschyi* for 24 and 48 hr and fractions of apoptotic cells were measured by flow cytometry method. Each experiment was performed in triplicate.

**Reactive oxygen species (ROS)** **detection**

The intracellular ROS levels were measured using DCFDA cellular reactive oxygen species detection kit according to the manufacturer's instructions (Abcam, Cambridge, MA). Briefly, 25000 cells were cultured overnight in a 96-well dark-sided culture plate. The cells were then stained with 100 μl of DCFDA solution in the dark at 37°C for 30 min. Next, cells were exposed to *D. kotschyi* (0-1600 µg/ml) for 4, 6, 8, and 24 hr. The fluorescence was quantified at excitation 490 nm and emission 520 nm using a Fluoroskan Ascent fluorometer (Thermo Scientific, Northumberland, UK). Each experiment was performed in triplicate (Rahimi et al., 2017 ▶, 2017b ▶).

**Gas chromatography/mass spectrometry (** **GC/MS** **)** **analysis**

To standardize the extracts, we analyzed the essential oils isolated from *D. kotschyi* using GC/MS (Table 1). GC/MS analysis was carried out by Agilent 5975 GC-MSD system. Hp-5MS column (30 m×0.25 mm, 0.25 µm film thickness) was used with helium as carrier gas with flow rate of 1.0 ml/min. The oven temperature was kept 20 min. at 50ºC and programmed to 280ºC at a rate of 5ºC/min, and kept 20 min. also constant at 280ºC for 5 min, at split mode. The injector temperature was 280ᵒC (at 20ᵒC/min). MS values were taken at 70 ev. Mass range was from 35 to 450 m/z. Identification was done based on the retention times compared to the authentic standards and by comparison of mass spectral fragmentation patterns (Ashrafi et al., 2017 ▶).

**Table 1:** Chemical compositions of essential oils of *D. kotschyi*

**% in oil**	**Retention index (RI)**	**Compound**	**% in oil**	**Retention index (RI)**	**Compound**
5.7 0.92 1.3 1.78 9.4 0.8 0.9 0.3 0.2 0.9 0.7 2.8 2.4 2.7 1.7 2.8 1.3	1113 1143 1182 1190 1405 1411 1418 1427 1436 1443 1452 14.61 1483 1496 1564 1574 1589	Neral Geraniol β-Himachalene Borneol β-Caryophellene Linalyl acetate Neryl propionate Caryophyllene oxide Δ-Cadinene α-Campholenal *Cis*-Verbenol *Trans*-Verbenol P-Mentha-1,5-dien-8-ol Terpinen-4-ol α-Terpineol *Trans*-Carveol α-Cubenene	0.98 7.2 0.6 2.7 0.8 6.7 1.3 0.72 3.82 0.5 15.8 2.8 2.7 1.2 3.5 1.8 4.2	918 939 952 969 972 984 991 1007 1016 1022 1038 1045 1062 1068 1073 1088 1095	α-Thujene α-Pinene Comphene β-Pinene Sabinene β-Myrcene β-Phellanderene Δ-4-Carene α-Terpinene P-Cymene Limonene β-*Trans*-Ocimene *Trans-*Sabinene hyd. *Cis*-Sabinene hyd. β-Thujone α-Terpinolene 1,8-Cineole

**Human lymphocytes isolation**

To investigate the effects of the extracts on normal cells, human lymphocytes were used. In this regard, 10 ml of the brachial vein whole blood was obtained from healthy subject and collected into a heparin-anti-coagulated tube. Lymphocytes were isolated using a Ficoll gradient as previously described (Askari et al., 2016b, 2018 ▶, 2019a ▶). In brief, blood cells were poured onto a Ficoll and centrifuged for 20 min at 400 g at controlled room temperature (15-25°C). The layer of buffy coat was carefully removed. For removing the residual red blood cells, the cells were co-cultured with 1x diluted RBC lysis buffer according to the manufacturer’s protocol and supernatant was aspirated without disturbing pellet. The live cells were counted using trypan blue solution 0.04% w/v, and cell viability and cell numbers were over 98% and 2.5×10^6^ cells/ml, respectively.

**Cell viability assay**

Cell viability was examined by colorimetric WST-1 assay kit according to the manual. In review, isolated lymphocytes were suspended in enriched RPMI-1640 and seeded at 10^5^ cell/100 µl per well in 96-well culture plates in triplicate. Each well was treated with different concentrations of the Soxhletextracts (0-1000 µg/ml) at final volume of 200 µl per well and incubated for 24, 48 and 72 hr. Next, the cells were incubated with 10% of final volume of each well with WST-1 reagent for 4 hr. Finally, the optical density was measured at 450 nm with the reference at 630 nm (Askari et al., 2016b ▶; Rahimi et al., 2017a ▶).

**Statistical analysis**

All results are shown as mean±SEM, and analyzed by Kruskal-Wallis’ test followed by Tukey's multiple comparison *post hoc* test (GraphPad prism software version 6). P values (p) were considered statistically significant, if they were lower than 0.05. 

## Results

**Phytochemical components of** ***D. kotschyi***

The phytochemical components of *D. kotschyi* essential oils were investigated extensively in our laboratory using GC/MS (Figure 5). GC/MS analysis results indicated the presence of monoterpenes and sesquiterpenes as dominant components of *D. kotschyi* (Table 1). For instance, the main monoterpene hydrocarbons were limonene, while geraniol was among the main oxygenated monoterpenes. Moreover, other phytochemicals recognized in *D. kotschyi* extracts were caryophyllene, verbenone, alpha-terpineol and perillyl alcohol.

***D. kotschyi*** **inhibits U87 cell viability**

To investigate the cytotoxic effect of *D. kotschyi* on glioblastoma cells, U87 cells were treated with increasing concentrations of *D. kotschyi* Soxhlet and soaked extracts (0-1600 µg/ml) for 24, 48 and 72 hr. Our results revealed that both extracts of *D. kotschyi* have cytotoxic effects on the U87 cells in both time- and dose- dependent manners. 

**Figure 1:**
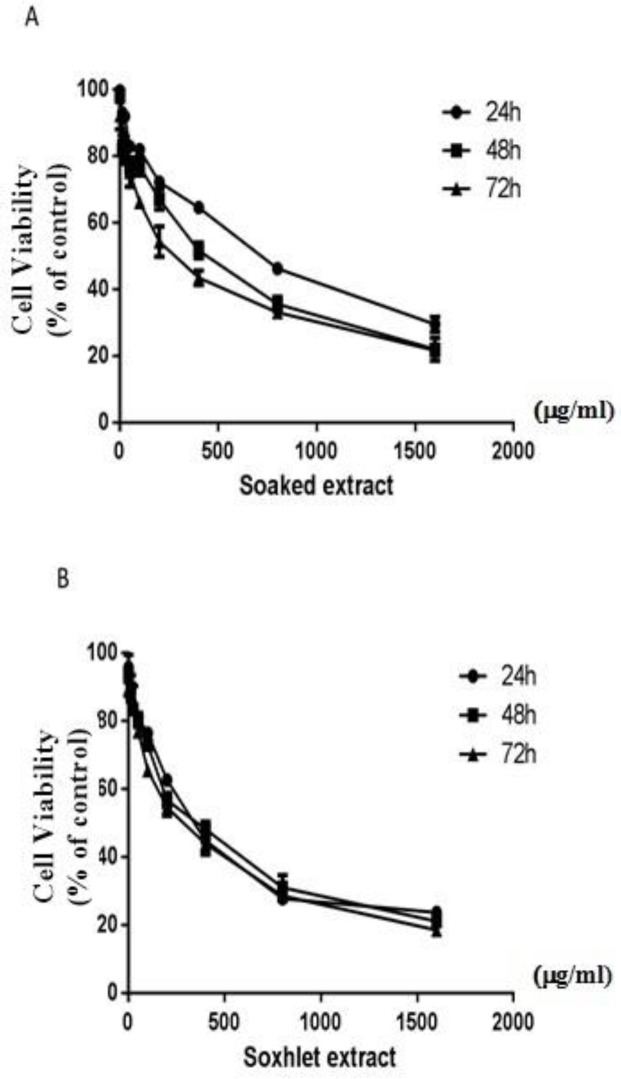
Growth inhibitory effects of *D. kotschyi *Soxhlet and soaked extracts on U87 cells. GBM U87 cells were treated with different concentrations of soaked (A) and Soxhlet (B) extracts of *D. kotschyi* for 24, 48, and 72 hr and the cell viability was determined by MTT assay. All data were obtained from three independent experiments (mean±SEM)

Following treatment with the soaked extracts, the IC_50 _values at 72, 48 and 24 hr were estimated about 286, 410 and 650 µg/ml, respectively (Figure 1A). Moreover, as shown in Figure 1B, the values of IC_50 _for the Soxhlet extracts at 72, 48, and 24 hr were 240, 306, and 334 µg/ml, respectively. 

***D. kotschyi*** **extracts induces cell death and apoptosis in U87 cells**

To further explore the regulatory effect of *D. kotschyi* on cell cycle progression in U87 cells, we performed the sub- G1 analysis using Flow cytometry method. Recent studies indicated that *D. kotschyi* induces cellular apoptosis in cancerous cells. In this line, PI staining method was used to investigate whether DNA fragmentation occurred in U87 cells in the presence of *D. kotschyi*. 

**Figure 2:**
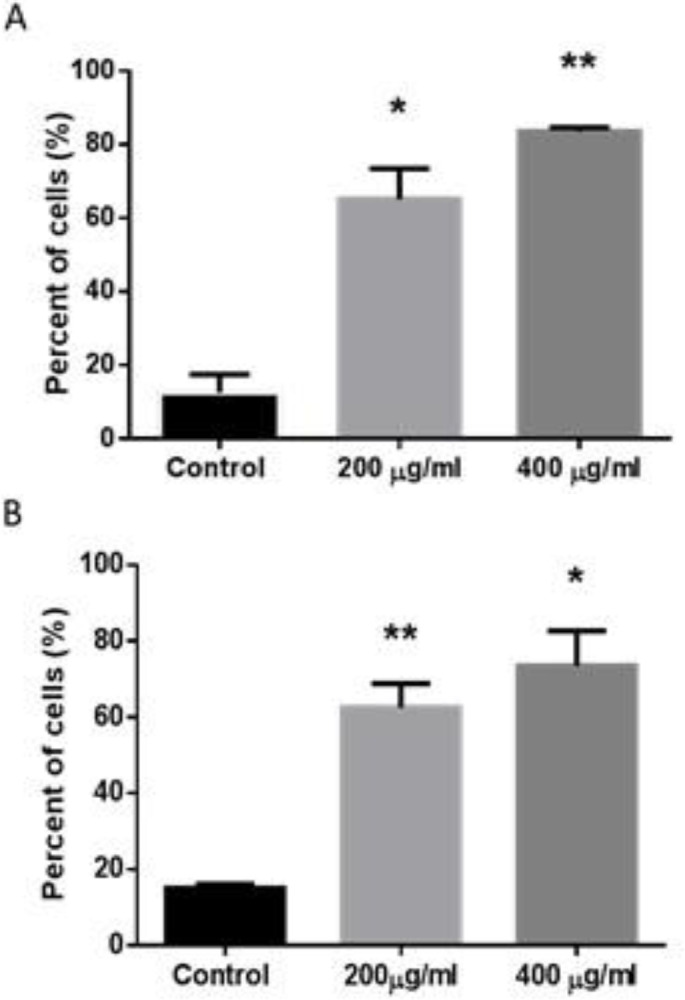
Effect of *D. kotschyi* Soxhlet extract on U87 cell cycle distribution. U87 cells were treated with different concentrations of *D. kotschyi* extracts for 24 (A) and 48 hr (B) and after PI staining were evaluated by flow cytometry for sub G1 DNA content. Columns, mean values obtained from three independent experiments; bars, SEM. *p<0.05 and **p<0.01

As shown in Figure 2, analysis of the sub-G1 region demonstrated that treatment with *D. kotschyi* enhanced cells population in sub-G1 phase resulting in cellular death. In the presence of *D. kotschyi* extracts for 24 hr, a total of ~ 60% of cells were observed in sub-G1 phase (Figure 2A), while the population of sub-G1 cells were increased after 48 hr treatment (Figure 2B). 

**Figure 3:**
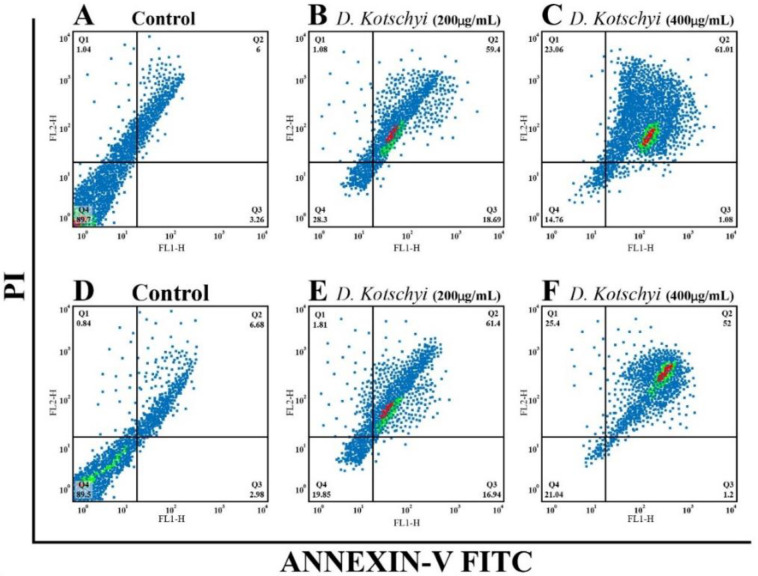
The apoptotic effects of *D. kotschyi* Soxhlet extract on U87 cells. U87 cells were treated with two different concentrations of *D. kotschyi* extracts for 24 (B, C) and 48 hr (E, F) and cellular apoptosis was analyzed using flow cytometry. All data were obtained from three independent experiments

To further verify our assumption that *D. kotschyi* extracts can induce cellular apoptosis, we studied the apoptotic effect of *D. kotschyi* on glioma cells by Annexin-V-FITC/PI staining. As shown in Figure 3, our data revealed that *D. kotschyi* extracts significantly promote U87 cell apoptosis in a dose- and time- dependent manner.

***D. kotschyi*** **induces ROS production in U87 cells**

**Figure 4:**
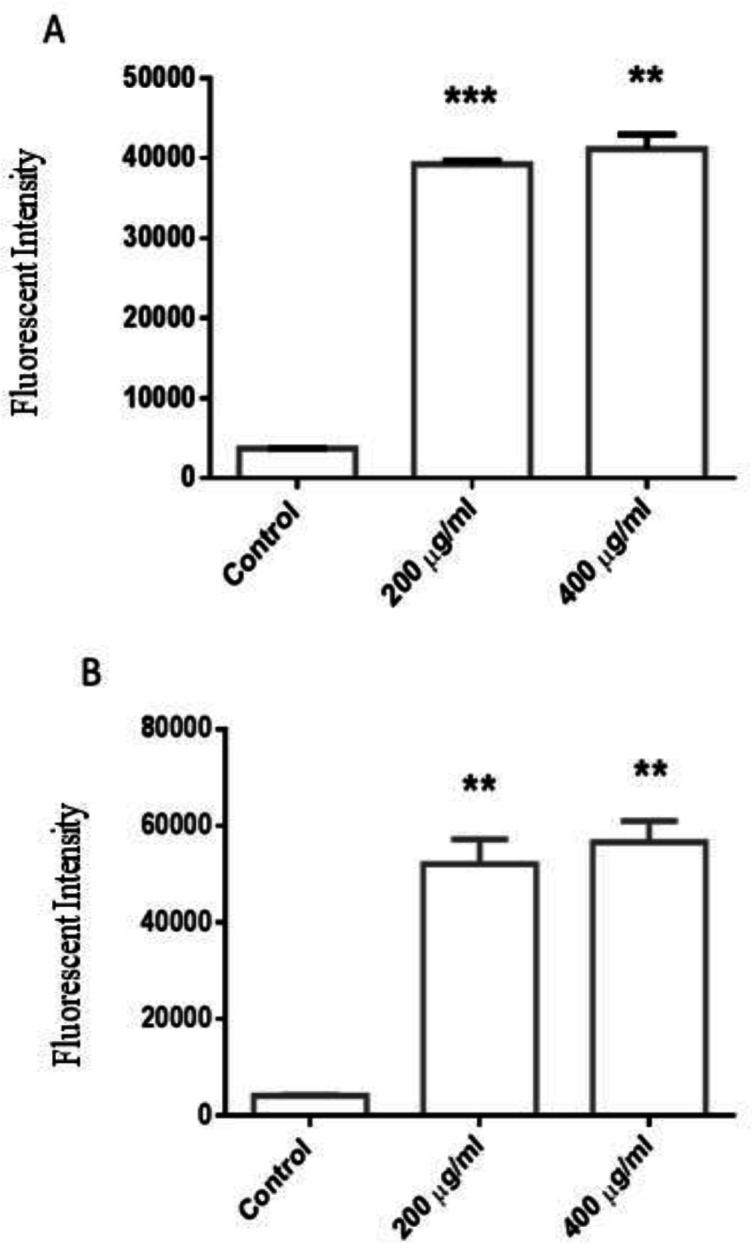
The effects of *D. kotschyi* Soxhlet extract on cellular reactive oxygen species. U87 cells were treated with *D. kotschyi* extracts for 4 (A) and 8 hr (B) and cellular ROS levels were measured by DCFDA assay. ROS: reactive oxygen species. Columns, mean values obtained from three independent experiments; bars, SEM. **p < 0.01 and ***p < 0.001

Generation of ROS is one of the main factors inducing cellular apoptosis. To gain better insight into anti-cancer mechanism of *D. kotschyi* extracts, we investigated their effects on ROS levels in U87 cells using the DCFDA assay. As shown in Figure 4, the Soxhlet extracts of *D. kotschyi* (200 and 400 µg/ml) significantly increased the DCFDA fluorescence intensity at both 4 and 8 hr after incubation in a dose- and time-dependent manner (p<0.001-0.01 for both cases).

**The effects of** ***D. kotschyi*** **extracts on normal cells**

To investigate the cytotoxic effect of *D. kotschyi* on normal cells, human isolated lymphocytes were treated with increasing concentrations of *D. kotschyi* Soxhlet extracts (0-1000 µg/ml) for 24, 48 and 72 hr. Our results revealed that both extracts of *D. kotschyi* have cytotoxic effects on the normal cell in both time- and dose- dependent manners. Following treatments with the Soxhlet extracts, the IC_50 _values at 72, 48 and 24 hr were estimated about 480, 690 and >1000 µg/ml, respectively (Figure 6). 

**Figure 5:**
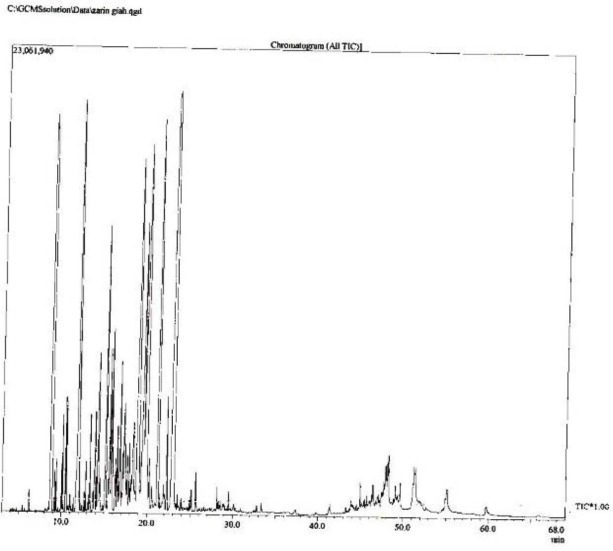
The chromatogram of essential oils of *D. kotschyi* obtained by GC/MS

**Figure 6:**
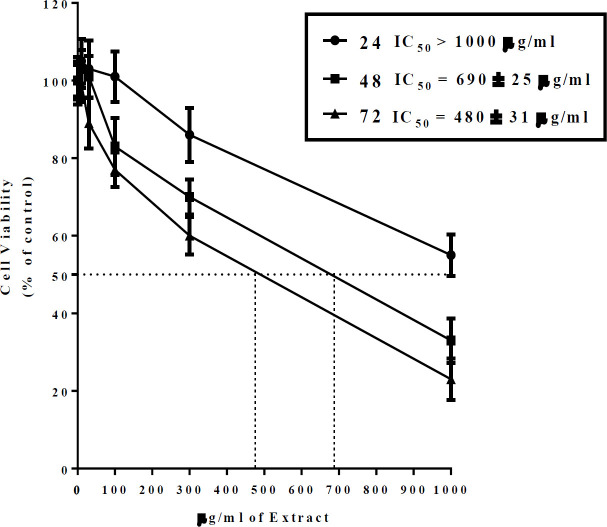
The effects of *D. kotschyi* Soxhlet extract on human isolated lymphocytes. The cells were treated with different concentrations of Soxhlet extracts of *D. kotschyi* for 24, 48, and 72 hr and then the cell viability was determined by WST-1 assay. (n=6, mean±SEM).

## Discussion

Cancer as a worldwide rising health problem is the second leading cause of death after heart disease. Glioblastoma multiforme (GBM) is one of the common primary brain tumors in adults accounting for approximately 80% of all gliomas (Omuro and De Angelis, 2013 ▶). Glioblastoma, as a more aggressive form of glioma, has a poor prognosis due to lack of effective clinical interventions (Zhang et al., 2012 ▶). Thus, new therapeutic agents are needed to improve the prognosis of patients.

In the present study, we examined the effect of *D. kotschyi* extracts on both U87 cellular proliferation and cell cycle progression. Our results indicated that following treatment with *D. kotschyi* extracts, U87 cells proliferation was inhibited in a time- and dose-dependent manner. Moreover, the cellular population in sub G1 phase was significantly elevated in comparison with the control group. In addition to evaluation of U87 cellular proliferation, we explored the apoptotic effects of *D. kotschyi* on glioma cells and our results demonstrated that *D. kotschyi* extracts induce cellular apoptosis in U87 cells. As shown in Figure 1, comparative studies indicated that the Soxhlet extracts exhibited more cytotoxic effects in comparison with the soaked extracts, on U87 cell growth. Therefore, we used Soxhlet extracts for further evaluations in our study.

It was revealed that tumor cells have higher level of ROS because of their increased metabolic activities (Panieri and Santoro, 2016 ▶). Several studies indicated that ROS plays key function in regulating several cellular process including cell growth, inflammatory responses, and cell death (Azad et al., 2009 ▶). To further investigate the cellular mechanism by which *D. kotschyi* extracts induce U87 cell death, we examined the intracellular ROS accumulation. We observed that different concentrations of *D. kotschyi* extracts induced significant elevations of ROS levels in the U87 cells, in a time-dependent manner which supports the hypothesis that possibly *D. kotschyi* induces cellular death through accumulating reactive oxygen species and oxidative stress. It was revealed that tumor cells have higher level of ROS because of their increased metabolic activities (Panieri and Santoro, 2016 ▶). Several studies indicated that ROS plays key role in the regulation of cellular process including cell proliferation, inflammatory responses, and cell death (Azad et al., 2009 ▶). Recent findings demonstrated that the effectiveness of some chemotherapeutic medications (e.g. doxorubicin) on cancer cell apoptosis may be correlated with the accumulation of intracellular ROS. In fact, in our study, we showed that the elevation of ROS levels at in the early hours may lead to inhibition of glioblastoma cell proliferation. In this regard, it was shown that accumulation of ROS in glioblastoma cells induced cellular apoptosis and autophagy through inhibition of PI_3_K/Akt/mTOR signaling pathway (Zhang et al., 2014 ▶). *D. kotschyi* is known in Iranian medicine as an analgesic and antispasmodic agent (Sadraei et al., 2015b ▶). Recent data showed that *D. kotschyi* contains several natural compounds including flavonoids which contribute to its anti-proliferative activity against tumor cells (Jahaniani et al., 2005 ▶). In this study, the anti-proliferative effects of *D. kotschyi* extracts were studied on both U87 cell growth and cell cycle progressionIt was indicated that b-caryophyllene (BCP) as one of the plant constituents with different anti-oxidant and anti-inflammatory effects (Askari and Shafiee-Nick, 2019b ▶, 2019c ▶), can exert cytotoxic effects against different cancers through accumulation of ROS, ceramides and cannabinoid type 2 (CB2) receptor (Legault and Pichette, 2007 ▶; Fidyt et al., 2016 ▶). Regarding the amount of BCP in the extracts, it seems that the cytotoxicity-induced in this line was partially mediated by BCP (~ 9.4% w/v).

In the present study, we also investigated the effect of Soxhlet extract on human isolated lymphocytes as normal cells. As a result, we found that following treatment with the Soxhlet extracts, the IC_50 _values at 72, 48 and 24 hr were estimated about 480, 690 and >1000 µg/ml, respectively (Figure 6). In fact, this finding showed that the concentrations of the extracts leading to cytotoxic effects in U87 gliblastoma cell line, are considerably lower than extractsthose that produce cytotoxicity against normal cells. However, it seems that further studies are still required to investigate the effects of the extracts on the viability of other normal cells. 

In conclusion, *D. kotschyi* induced U87 glioblastoma cell death is accompanied by ROS generation, which presented *D. kotschyi* as a promising anticancer plant against glioblastoma multiform (GBM). Further studies are needed to understand the molecular mechanisms by which *D. kotschyi* induced cancer cell death in human malignancies.
